# Longwise Cluster Analysis for the Prediction of COVID-19 Severity within 72 h of Admission: COVID-DATA-SAVE-LIFES Cohort

**DOI:** 10.3390/jcm11123327

**Published:** 2022-06-10

**Authors:** Rodrigo San-Cristobal, Roberto Martín-Hernández, Omar Ramos-Lopez, Diego Martinez-Urbistondo, Víctor Micó, Gonzalo Colmenarejo, Paula Villares Fernandez, Lidia Daimiel, Jose Alfredo Martínez

**Affiliations:** 1 Precision Nutrition and Cardiometabolic Health Researh Program, Institute on Food and Health Sciences (Institute IMDEA Food), 28049 Madrid, Spain; victor.mico@imdea.org (V.M.); jalfredo.martinez@imdea.org (J.A.M.); 2 Biostatistics & Bioinformatics Unit, Madrid Institute for Advanced Studies (IMDEA) Food, CEI UAM + CSIS, 28049 Madrid, Spain; roberto.martin@imdea.org (R.M.-H.); gonzalo.colmenarejo@imdea.org (G.C.); 3 Medicine and Psychology School, Autonomous University of Baja California, Tijuana 22390, Baja California, Mexico; oscar.omar.ramos.lopez@uabc.edu.mx; 4 Internal Medicine Department, Hospital Universitario HM Sanchinarro, 28050 Madrid, Spain; dmurbistondo@gmail.com (D.M.-U.); pvillares@hmhospitales.com (P.V.F.); 5 Nutritional Control of the Epigenome Group, IMDEA Food Institute, CEI UAM + CSIC, 28049 Madrid, Spain; lidia.daimiel@imdea.org; 6 CIBERobn Physiopathology of Obesity and Nutrition, Institute of Health Carlos III (ISCIII), 28029 Madrid, Spain

**Keywords:** COVID-19, Charlson Comorbidities Index, cluster analysis, longitudinal cluster, individualized management

## Abstract

The use of routine laboratory biomarkers plays a key role in decision making in the clinical practice of COVID-19, allowing the development of clinical screening tools for personalized treatments. This study performed a short-term longitudinal cluster from patients with COVID-19 based on biochemical measurements for the first 72 h after hospitalization. Clinical and biochemical variables from 1039 confirmed COVID-19 patients framed on the “COVID Data Save Lives” were grouped in 24-h blocks to perform a longitudinal k-means clustering algorithm to the trajectories. The final solution of the three clusters showed a strong association with different clinical severity outcomes (OR for death: Cluster A reference, Cluster B 12.83 CI: 6.11–30.54, and Cluster C 14.29 CI: 6.66–34.43; OR for ventilation: Cluster-B 2.22 CI: 1.64–3.01, and Cluster-C 1.71 CI: 1.08–2.76), improving the AUC of the models in terms of age, sex, oxygen concentration, and the Charlson Comorbidities Index (0.810 vs. 0.871 with *p* < 0.001 and 0.749 vs. 0.807 with *p* < 0.001, respectively). Patient diagnoses and prognoses remarkably diverged between the three clusters obtained, evidencing that data-driven technologies devised for the screening, analysis, prediction, and tracking of patients play a key role in the application of individualized management of the COVID-19 pandemics.

## 1. Introduction

The Severe Acute Respiratory Syndrome Coronavirus 2 (SARS-CoV-2) appeared around December 2019 in Wuhan (China) and has been spreading all around the globe thenceforth [1,2]. The World Health Organization (WHO) declared the disease (COVID-19) caused by SARS-CoV-2 as a pandemic in March 2020, based on the incidence growths due to the high contagiousness and high levels of lethality presented [3]. The major challenge for clinicians and practitioners has been the wide clinical presentation form of the disease and requiring the decision of intensive care unit (ICU) admission, together with the use of mechanical ventilation. Patients with COVID-19 could present as asymptomatic or with milder symptoms (including fever, sore throat, dry cough, dyspnea, myalgia, headache, or diarrhea) or with more severe symptoms, such as chest pain, hypoxemia, pneumonia, and other complications [4]. Since the appearance of this pandemic, several authors have tried to stratify the patients depending on the symptoms, oxygen saturation, or the chest computed tomography in order to predict the severity of the patients, aiming to facilitate decision making in the clinical practice [5]. 

COVID-19 infection displays a mean incubation period between 6 and 7 days from the initial infection, followed by a viremic phase from the 8th day to the 10th day. However, the delay between symptom onset and hospitalization could vary from 2.6 to 9.7 days, depending on the country and the age of patients [6]. The delay of detection and hospitalization has a large impact on the concurrent inflammatory stage, and, thus, on the prognosis and the fatality of the disease [7]. These manifestations are accompanied by microvascular damages caused by the cytokine “storm” [8], and often, the pathophysiological COVID-19 condition is also associated with bacterial infections [9] and with body metabolic impairments [10,11], where prescribed anti-inflammatory medications also may play a role [12].

With all of this, the use of routine laboratory biomarkers is the key monitoring tool to predict the prognosis of the disease. There are several studies that have focused their research on a limited number of these markers or have uniquely performed cross-sectional analyses at the baseline and their relationship with the prognosis of these patients [13,14]. Thus, the identification of patients that are more likely to develop severe illness after diagnosis is a critical *checkpoint* in order to decrease mortality rates, as well as to avoid the collapse of medical care within the hospitals [15]. Therefore, taking into account the time evolution of comorbidities and potential organ injuries throughout the course of severe COVID-19 is crucial in the precise clinical management of patients, influencing treatment approaches and recovery rates [16] where inflammation has a very strong role [17], as well as immunity and hematological alterations [18] and liver dysfunctions [19]. All of this emphasizes the need for a clustered clinical management of this disease and one that would lead to achieve more personalized and effective interventions [20].

In this regard, understanding the short-term longitudinal variation and the specific profiles of these biomarkers based on the severity of disease progression would allow the development of stratification tools [21] to characterize distinctive phenotypes concerning patients with COVID-19 that predict their potential prognosis [22]. In this regard, the use of data science methods to identify underlying patterns or profiles present in patients with COVID-19 could shed light on the mechanisms that occur and would allow for the prescription of personalized treatments through the determination of clusters of patients attending objectively measured variables [5]. Based on this background, the present study aimed to explore data from patients admitted to all HM private hospitals in the Madrid region during the first pandemic peak reported in Spain, in order to find clusters of patients based on the biochemical measurements for the first 72 h of attendance and further implication in their prognosis.

## 2. Materials and Methods

### 2.1. Patients Database

The data used for the present analysis were framed on the “COVID Data Save Lives” (COVIDDSL) initiative carried out by the *HM Hospitales.* This initiative made freely available an anonymous dataset containing the information from the Electronic Health Record (EHR) system of the HM Hospitales (information available at https://www.hmhospitales.com/coronavirus/covid-data-save-lives/english-version (accessed on 20 July 2020)). The anonymized information contains the records of 2310 patients that were admitted with a diagnosis of COVID-19 between 26 December 2019 and 10 June 2020. Multicenter longitudinal information from this EHR comprise different datasets corresponding to the main clinical characteristics of different domains. Each patient was identified by an anonymized unique admission code. The datasets include information about the COVID-19 treatment process, including complete information on admission and diagnoses, treatments, ICU admissions, diagnostic imaging tests, laboratory results, drug administration, and cause of discharge or death). This study was conducted according to the guidelines of the Declaration of Helsinki and approved by the Ethics Committee of the HM hospitals consortium (CEI HM Hospitales Ref No. 20.05.1627-GHM).

### 2.2. Data Collection and Definitions

The data sets were preprocessed considering only adult patients with confirmed COVID-19. Both clinical and biochemical variables were selected and grouped in blocks of 24 h to 72 h from the patients’ admission to the hospital. In those patients that presented more than one measure per day, the median value was used to avoid the potential effect of extreme values for these variables. Additionally, patients were categorized according to the cause of discharge or admission to the ICU and the administration of mechanical ventilation. Reported death and mechanical ventilation variables were used to test the prognostic value of the current exploratory analysis.

Data included for the exploratory analysis were patient’s age, sex, clinical history of previous diseases, vital signs and tests performed throughout the hospitalization, and the medications administered until the discharge. The vital sign variables included for the analysis were oxygen saturation (%), body temperature (°C), heart rate (beats/min), and systolic and diastolic blood pressure (mmHg). The following parameters were selected from the different tests collected: white cell proportions including leukocytes (1000/µL), basophil (%), eosinophils (%), lymphocyte (%), monocyte (%), and neutrophils (%); red cell markers including red cell distribution width (RDW, in %), hemoglobin (g/dL), hematocrit (%), mean corpuscular hemoglobin (pg/cell), mean corpuscular hemoglobin concentration (g/dL), and mean corpuscular volume (fL); platelets and prothrombin markers such as mean platelet volume (%), platelet count (1000/µL), the international normalized ratio (INR), prothrombin activity (%), and prothrombin time (seconds); metabolic markers and electrolytes including glucose (mg/dL), Gamma-glutamil transferase (GGT, in IU/L), aspartate aminotransferase (AST, in IU/L), alanine aminotransferase (ALT, in IU/L), sodium (mmol/L), and potassium (mmol/L); and finally, inflammatory and catabolic markers such as C-Reactive Protein (CRP, in mg/L), D-Dimer (ng/mL), lactate dehydrogenase (IU/L), creatinine (mg/dL), and urea (mg/dL). 

Additionally, International Statistical Classification of Disease and Related Health Problems (ICD-10) coding tables with clinical records of diseases and procedures, as well as medications classified by ATC5/ATC7, for each patient and time point were also condensed in categories and activity of medications, respectively. The coded information was used to carried out complementary descriptive analysis. Additionally, clinical variables were encoded following the criteria of the Charlson comorbidity index (CCI) categories [23] to adjust the logistic regression models and measure the effect in the models of concomitant diseases as a potential confounder in the prognosis of these patients.

### 2.3. Statistical Analysis

Patients with less than 50% of missing values for the selected variables during the first 4 blocks of 24 h were selected to conduct the present analysis. Patients were categorized, by the median number of comorbidities at the baseline (by CCI), patients with 3 or less comorbidities and those with more than 3 comorbidities at the baseline, to carry out the descriptive analysis, including means and standard deviations (SD) for quantitative variables and absolute value with percentages for categorical variables. Student’s t tests for continuous variables and chi-squared tests for categorical variables were used to assess differences between patients from both comorbidity groups.

The longitudinal unsupervised clustering was performed by using the *Kml3d* library, which provided a longitudinal implementation of the widely used k-means algorithms [24]. The technique used for this study was an unsupervised non-parametric cluster analysis that classifies the trajectories of the patients by simultaneously providing the 33 routine biochemical parameters from the first 72 h after the admission of the patients. This technique implements a path expectation-maximization algorithm by alternating different initialization methods to obtain the most stable solution for the clusters, and it can feature groups of patients associated with specific disease risks. Clustering approaches are not ultimately predictive, but they are descriptive and contribute to identify patterns concerning hidden structural data, which do not demand a formal hypothesis. Indeed, clustering analysis can feature groups of patients associated with specific disease risks. Clustering permits targeting patients in a cost-effective feasible nature and relevant clinical impact. Cluster analysis has been used to characterize risk factors associated with diseases [25] and may require further regression analysis to predict other related variables [26]. This library was used to specifically cluster patients based on the joint trajectories of the selected clinical and biochemical variables throughout the 24-h time periods during the first 72 h of hospitalization. A range of 2 to 10 clusters was assayed to fit the most adequate solution for the model, based on the lowest Bayesian information criterion (BIC) and the clinical relevance of clustering solutions (measured by the severity of outcomes related to the cluster using logistic regression), resulting in a final best solution of 3 clusters. Principal component analysis was conducted to visualize the categorization of the patients. The relative importance of the variables and the time periods was estimated through variable/time permutation to gain a better understanding of the most important variables and times in the clusters obtained. ANOVA analysis was carried out to compare clinical characteristics among clusters, and a Tukey *post hoc* analysis was applied to compare individual groups.

A multivariable logistic regression model was used afterwards to estimate the gain upon inclusion of the clusters previously obtained as independent variables for the prediction of two outcome variables, namely death and administration of mechanical ventilation during hospitalization. Three different models were developed to evaluate the effect of the inclusion of the cluster assignment, in addition to the main factors that impacted the COVID prognosis. Model 1 used age-independent CCI, sex, and age as predictor variables; model 2 was additionally adjusted by temperature and oxygen saturation at admission; and the final model 3 was additionally adjusted by the cluster assignments. Area under the curve (AUC) from receiver operating characteristic curves (ROC) was estimated to evaluate the predictive value of each model. All the statistical analyses were performed using R statistical software version 4.0.1 (R Project for Statistical Computing) within RStudio statistical software version 1.4 (Rstudio Team. Rstudio: Integrated Development Environment for R. Boston, MA, USA).

## 3. Results

### 3.1. Study Sample Description

The cleaned dataset (Table 1) contained 1039 confirmed COVID-19 patients, 60% male and 40% female, with a global age mean of 68.5 years. The mean days of hospitalization were 10.1, with 5.4% of the patients admitted to ICU and 62.6% receiving mechanical ventilation during the hospitalization. The main cause of medical discharge was home referral (78.5% of patients), while the referral to other centers corresponded to 6.2% of the hospitalization, and death represented 11.5% of the patients. The patients presented an average CCI of 3.6 at hospitalization. As expected, when the patients were categorized by CCI with a cut-off of 3 points (Table 1), those above the cutoff were older and evidenced worse health status concerning hospitalization features and higher death, and they suffered more comorbidities including cardiovascular events, liver diseases, diabetes, and cancer; however, a significant association with sex was observed.

### 3.2. Patient Clusterization

The cluster analysis was developed to categorize the sample based on the longitudinal evolution of multiple vital signs and laboratory tests (see Section 2). The best clustering was obtained with three clusters. Supplementary Figure S1b displays a PCA with all these variables, colored by the three clusters obtained. We can see the good separation of the patients achieved by this longitudinal clustering. In addition, in order to interpret the clustering, we estimated the relative importance of the different variables and times by permutation-based feature/time importance analyses. The resulting ranked importance of variables and times are displayed in Supplementary Figure S1c, where it can be seen that monocytes, GGT, neutrophils, prothrombin time, and urea were the most remarkable variable contributors to the clustering, and the first 24 h is the most important time of all.

In addition, in Table 2, we analyzed the association of these clusters with different baseline and outcome variables, in order have an idea of the clinical profiles of the three clusters. In this way, Cluster A encompassed patients with lower hospitalization, ICU stay, and clinical complication rates, displaying a death rate of only 1.6%; Cluster B showed an intermediate prevalence of chronic diseases with a fatality incidence of 14.4%; and Cluster C showed the eldest group of patients, with a mortality rate of 37.4% and a higher clinical morbidity prevalence. 

**Figure 1:**
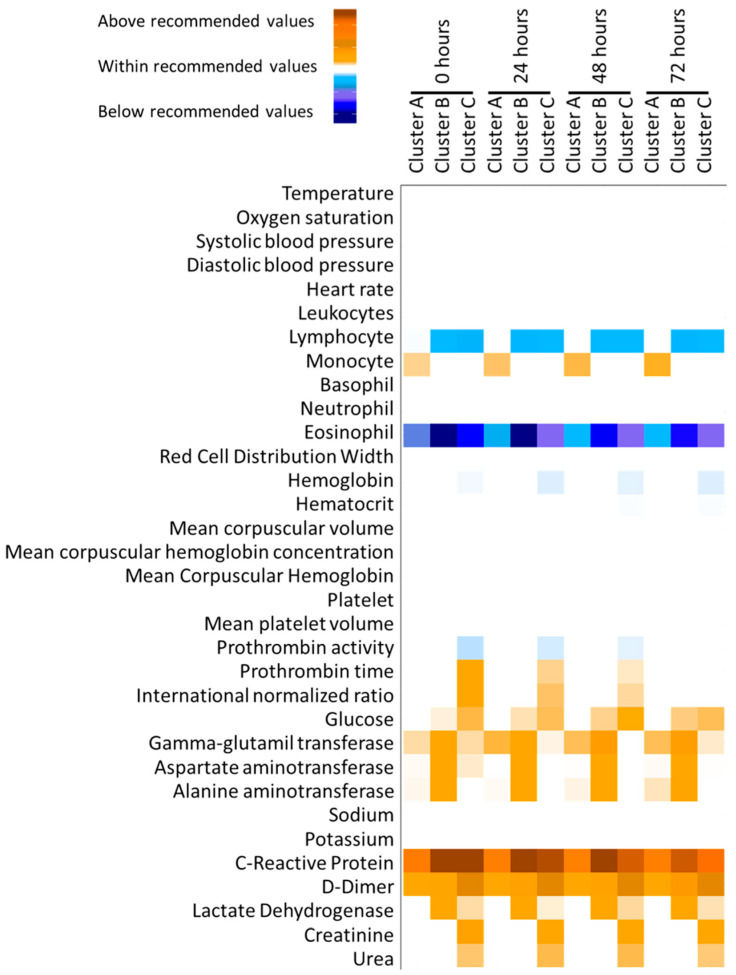
Heatmap plot of adequacy to reference values for clinical variables included in the cluster analysis. White means that the mean value for the cluster was within the recommended values; meanwhile, blue and orange intensity represent the deviation from the recommended values below and above, respectively.

Additionally, clinical variables evolved during the initial 72 h after hospital admission according to different cluster profiles, as can be seen in Figure 1, where the time evolution of these variables is displayed for the three classes, color coded in reference to recommended values (above, within, below). In general, Cluster C presented the most altered medical variables in comparison with the other two clusters, while Cluster B showed a mildly severe inflammatory condition. More specifically, the patients in Cluster B showed the lowest eosinophil levels and the highest levels of GGT, AST, ALT, C-reactive protein, and lactate dehydrogenase during the 72 h compared to the other clusters. Meanwhile, Cluster C presented the lowest lymphocyte levels and prothrombin activity, as well as the most elevated levels for prothrombin time, INR, glucose, D-dimer, creatinine, and urea (Figure 1).

Vital signs (Supplementary Figure S2) indicated that, while Cluster A presented less unhealthy symptoms, Cluster B and C displayed significantly worse clinical outcomes maintained throughout all time points (0–72 h). When white blood cell count was observed (Supplementary Figure S3), Cluster A involved fewer biological abnormalities. Specifically, lower levels of eosinophils were detected in the three clusters at all-time points, while only Cluster B and Cluster C had lymphocyte counts below the laboratory references. Curiously, Cluster A presented high levels of monocyte count. Those cluster differences were present across the 72-h measured course (Figure 1 and Supplementary Figure S3). Red blood cell levels, despite some significant cluster differences, were not different to normalized values (Figure 1 and Supplementary Figure S4).

Regarding blotting (prothrombin activity and time besides international normalized ratio) and hepatic related enzymes (ALT, AST, and GGT), Cluster C had altered high levels of those indications, while the other two cluster were closer to reference normality (Figure 1 and Supplementary Figures S5 and S6). Finally, inflammation (C-reactive protein) and thrombosis (D-Dimer) examinations, as well as lactate dehydrogenase, were impaired in all the clusters, with a greater severity in Cluster B and C compared to Cluster A, while renal functionality assessed by creatinine and urea were only altered in Cluster C (Figure 1 and Supplementary Figure S7).

### 3.3. Logistic Regression Models to Predict Severe Outcomes

**Figure 2:**
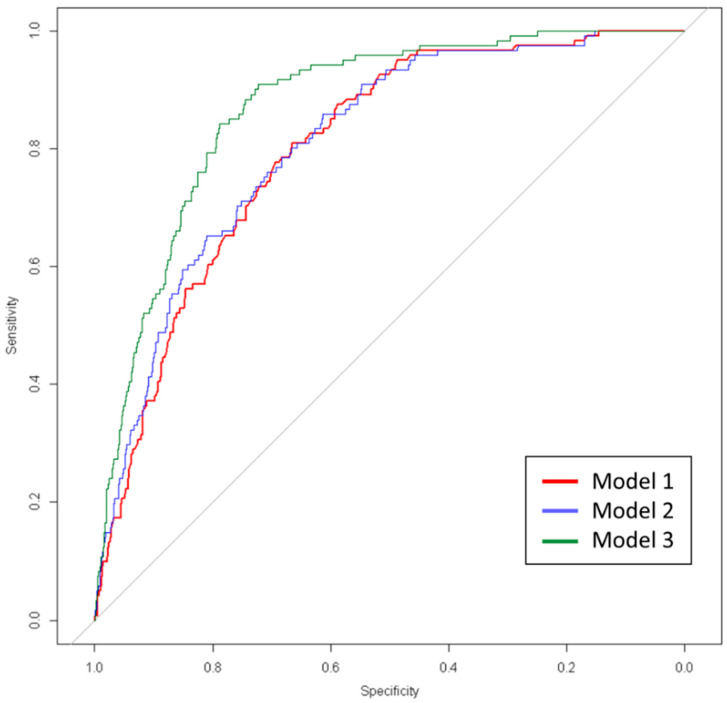
ROC curve of logistic regression for the three models.

Finally, a logistic regression model was fitted to discern the capacity of the modeled clusters to predict the disease fatality (Table 3, Figure 2). The first model, including the age-independent CCI, sex, and age as predictors, showed only age and sex with significant *p*-values and an AUROC of 0801. A second model, which added oxygen saturation and temperature (the former significant but not the latter) to the previous one, had a negligible increase of AUROC to 0.81. However, the inclusion of the cluster variable in the third model (green line in Figure 2) resulted in a large boost of the AUROC, up to 0.87. The third model presented the highest value of AUC, showing the better capacity of death prediction (*p*-value obtained by parametric bootstrapping for differences between Model 1 vs. Model 3 and Model 2 vs. Model 3, <0.001 and <0.001, respectively).

**Figure 3:**
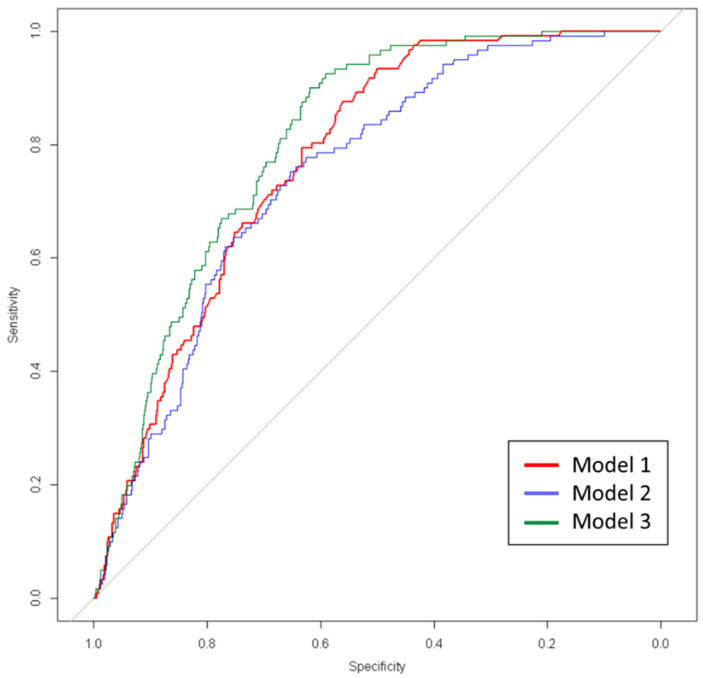
ROC curve of logistic regression for the three models.

A similar analysis was performed to predict the risk of mechanical ventilation using these models (Table 4). The obtained results were similar, with a better predictive capacity in Model 3 compared to the other two models (Figure 3), confirming the utility of patients’ clusterization (*p*-value obtained by parametric bootstrapping for differences between Model 1 vs. Model 3 and Model 2 vs. Model 3 = 0.023 and <0.001, respectively).

## 4. Discussion

Coronavirus disease has affected all nations and territories, while several investigations are now being conducted to seek personalized clinical prescriptions and provide epidemiological surveillance to control this pandemic [15,27]. Indeed, research concerning the early symptomatic identification and assessing specific traits involving clinical manifestations, medical outcomes, and epidemiological estimates with machine learning models offers huge opportunities for precision medicine despite some limitations and challenges [28]. In this context, the COVID-19 disease presents a unique prospect to understand whether there are distinct phenotypes of COVID-19 outcomes, whose knowledge will provide important benefits not only for the personalized management of infected patients, but also for optimizing health care systems and for devising public health policies [29] by considering phenotypical plus family and clinical history backgrounds, as well as individual lifestyle factors [30].

The implementation of multivariate statistical and bioinformatic instruments to provide valid information for clinical purposes includes hierarchical cluster analysis, principal component analysis, random forest, discriminant analysis, support vector machine algorithms, and neural network-based deep learning methods, with value on disease characterization, diagnosis, and treatment [20]. In this context, a longitudinal cluster analysis was implemented on the “COVID Data Save Lives” (COVIDDSL) dataset to unhidden statistically significant clinical variables and the internal structure, as performed elsewhere with COVID-19 infected patients [31].

In our clinical setting, regarding a group of Spanish public/private hospitals, applying longitudinal cluster analyses enabled three distinctive COVID-19 medical phenotypes to emerge: Cluster A characterized by including patients’ mild inflammatory symptoms and low death occurrence (1.6%), Cluster B featuring important immune-inflammatory distress and specific liver dysfunctions with a rate of 14.4% mortality, while Cluster C encompassed specific coagulation disorders and renal alterations, in addition to inflammatory and immunocompetence abnormalities with a fatality prevalence of 37.4% of the patients. Thus, survival times across clusters notably differed in the three groups of patients, which is key for ameliorating disease management and outcomes by considering individualized patient profiling, predictive personalized models, and precision cost-effective risks, alleviating procedures as previously described in the palliative treatment of liver tumors using unsupervised artificial intelligence [32]. Moreover, the age and number of comorbidities, as associated with increased risk of mortality in patients with COVID-19, need to be accounted for [33], as delineated in the three A, B, and C clusters.

In this scenario, analyses concerning longitudinal COVID-19 disease trajectories were able to recognize vulnerable population clusters that would particularly benefit from specific health resources and provide insights for public health targets in order to manage the COVID-19 infectious pandemic. Thus, tuberculosis and HIV/AIDS, hepatitis, cardiomyopathies, and diabetes were consistently associated with an increased risk to be found in a more vulnerable cluster [34]. Furthermore, a comprehensive measurement of dysfunction severity of six organ systems based on the Sequential Organ Failure Assessment (SOFA) score revealed that cardiovascular, central nervous system, coagulation, liver, renal, and respiration pathobiology were able to identify distinct strata of COVID-19 patients, as defined by the baseline post-intubation SOFA. This includes findings suggestive of inflammation as a mechanism involving differential COVID-19 disease severity outcomes, as well as a heterogeneous physiopathological lung illness [29], which is in accordance with some of our findings, given that inflammatory responses, clothing, hepatic/renal alterations, and impaired immunocompetence were markers involved in cluster discrimination 

Another study developed with machine learning tools and based on a decision tree model to anticipate COVID-19 outcomes from a list of 132,939 recovered COVID-19 subjects evidenced that mortality prevalence was specifically clustered among males, older cases, and hospital admission history as predictors of case fatality [35]. In addition, a database study encompassing hospitalized COVID-19 patients over 24 and 48 h in the Mount Sinai Health System predicted intubation, intensive care unit transfer, and mortality and was able to identify important features, such as pulse oximetry with clinical importance in the outcome [36]. Results from the current analyses confirm trends during the 72-h outcomes among the three clusters, with some differential responses concerning PCR, hemoglobin, and coagulation indicators, while the fitted logistic regression model for the risk of mechanical ventilation and death considered both variables independently influenced by cluster allocation.

Another analysis devised to generate an accurate diagnosis model of COVID-19 based on routine tests and clinical symptoms by applying machine learning to COVID-19 data found several associations between clinical variables, such as having idiosyncratic levels of circulating lymphocytes and neutrophils, suggesting that COVID-19 patients could be clustered into several phenotype subtypes based on immune cells, gender, and declared symptoms, which could overcome the influence of a low testing capacity or the concurrent impact of other bacterial or viral infections [37]. Indeed, our cluster model demonstrated discrimination abilities associated with lymphocyte, monocyte, and eosinophil counts among then and during the 72 h after hospitalization.

Noteworthy, anemia and iron deficiency may play a role in the Coronavirus disease, as shown in a systematic review and associated meta-analyses, where hemoglobin levels were lower with older age but higher in subjects with diabetes, hypertension, and overall comorbidities and those admitted to intensive care [38], which is independently categorized by Cluster C in our model

The severe proinflammatory state commonly reported in COVID-19 patients has been associated with the activation of coagulation pathways and thrombosis [39], as well as by a characteristic coagulopathy and procoagulant endothelial phenotype [40]. The current clustered model for COVID-19 patients classified prothrombin activity and time, specifically in Cluster C, and also demonstrated some stratification competences in Dimer-D measurements, but not in increased platelet consumption. Interestingly, thrombocytopenia is relatively uncommon in COVID-19, being estimated that the dysregulated immune system responses as coordinated by inflammatory cytokines, lymphocyte cell death, and endothelial damage are involved [41]. Thus, patients with COVID-19 may suffer coagulation and thrombotic abnormalities, stimulating a hypercoagulable condition and increasing thromboembolic incidence [42].

Associations between blood biomarkers such as the neutrophil-to-lymphocyte ratio with the severity of COVID-19 lesions have been established, as well as with other specific and unspecific proinflammatory markers, such as CRP and other measures commonly analyzed for COVID-19, such as hemoglobin, D-dimers, and eosinophils counts [18], which should orientate the clinician for infected patients’ management being eased by the existence of algorithms and cluster categorization. Further statistical analyses indicated that inflammatory CRP and D-dimer levels were increased and can assist as early indicators of severe COVID-19 cerebrovascular problems [27].

In these circumstances, exacerbated innate and adaptive immune responses are crucial in foreseeing the development and progression of NAFLD in COVID-19 patients [19]. A specific implication of severe COVID-19 in NAFLD patients putatively mediated by immunocompetence status is highlighted in the B cluster, where transaminases and liver health markers showed abnormal values and may drive personalized medicine approaches, as prompted by the allocation to a cluster with related measurements uncovering therapeutic targets. In a previous report, patients concerning this COVID-DATA-SAFE-LIFES cohort were categorized following conventional criteria to explain disease severity and deaths, which verified that liver and proinflammatory features are important determinants of COVID-19 morbidity and mortality in order to ameliorate the understanding of morbid manifestations of COVID-19, besides to help the therapy decision-making protocols under a personalized medicine scope [11]. Indeed, the liver health and coagulation axis appears as a relevant surrogate for elucidating some COVID-19 outcomes linked to systemic inflammation [43], as well as thrombotic and fibrinolytic disturbances [44], which were deciphered in the currently emerged three clusters, including some markers of global health such as lactate dehydrogenase or creatinine/urea measurements [45], as particularly discriminated in Cluster C. Interestingly, hemoglobin and prothrombin values evidenced divergent patterns after the following 72-h period, which represent a worth for a cluster monitor. Indeed, our results provide a tool in the early management of COVID-19 patients, in contrast to other related papers in COVID where it has been taken into account with cardiac biomarkers [46] or other more complex techniques, such as imaging-based prognosis or gene/protein expression [47,48].

This research had some limitations and strengths. Thus, as a multipurpose cohort, the aims and hypotheses were assigned after the database was closed, and this was partly overcome by the large number of collected clinical determinations and the relatively high sample size. In addition, the initial uncertainties about the clinical management guidelines and concurrent morbid conditions/medications in COVID-19 patients may have an impact on data interpretation, although we provided information about pharmacological treatments (Supplementary Table S2) and several diseases at admission. 

The identification of subgroups of COVID-19 patients through the longwise cluster analysis performed in this study allowed the identification of latent profiles of COVID-19 patients to shed light on the most appropriate treatment focused on objective routine blood markers commonly used in clinical practice, unlike other articles that only study a single marker follow-up [27], cross-sectional analyses [14], composite index [29], or non-objective markers [13]. Moreover, a model using machine learning was able to predict case fatality in the elderly population, with a large history of hospital admission, which increases the rate of COVID-19 death [35]. Novel aspects of this analysis concerned the discrimination of patients by clustering routine determinations and being able to forecast death rates and associated comorbidities in the first 72 h. Previous studies have focused on exploring the value of these bioinformatic tools for coronavirus diagnosis and treatment [20], including image processing [49]. These results have been reinforced in systematic and metanalysis, which described clinical subgroups, while other researchers using result-driven technologies implemented the screening, analyses, and predictors of data tracking to confirm death cases [50]. Furthermore, the longitudinal follow up for 72 h allowed the confirmation of trends and alignments, giving support to the interest of multiple clinical analytical measurements at entrance. Actually, healthcare provision necessitates the backing of innovative skills and strategies, including artificial intelligence (AI), Big Data, and machine learning approaches to combat and project actions against new diseases such as COVID and other complex syndromes. Identifying the pool of cases and predicting where this viral infection and associated comorbidities will move in future interventions require collecting clinical information and bioinformatically analyzing available preceding data [50].

## 5. Conclusions

Summing up the current cohort, by applying a longwise cluster analysis of the first 72 h enabled to materialize three discriminated COVID-19 clinical clustered phenotypes: Cluster A, featuring patients mainly displaying mild inflammatory abnormalities and a low fatal occurrence below 2%; Cluster B, involving specific immune-inflammatory and explicit liver dysfunctions, with a mortality incidence around 15%; and Cluster C exhibiting hemoglobin, prothrombin, and renal impairments, together with importantly altered inflammatory and immune responses, resulting in about 40% of deaths in this group. Indeed, patient diagnoses and prognoses remarkably diverged in the three clusters, which is relevant for considering predictive patient alignment, tailored precision clinical prescriptions, personalized cost-effective engagements, and alleviating epidemiological measures, as pioneers reported in diverse communicable and non-communicable diseases using artificial intelligence and machine learning instruments. Actually, medical-driven technologies devised for the proper screening, analysis, prediction, and tracking of SARS-CoV-2 infected patients are partaking significant developments and applications for the precision and individualized management of the COVID-19 pandemics.

## Figures and Tables

**Table 1:** Baseline and outcome characteristics of COVID-19 patients from DATA SAVE LIVES categorized by the Charlson comorbidity index.

	Overall	≤3 Points	>3 Points	*p*
n	1039	533	506	
Age	68.5 (15.5)	58.0 (11.9)	79.5 (10.3)	<0.001
Sex (male (%))	626 (60.3)	328 (61.5)	298 (58.9)	0.419
Hospitalization (days)	10.1 (8.6)	8.9 (7.7)	11.3 (9.3)	<0.001
ICU stay (yes (%))	56 (5.4)	29 (5.4)	27 (5.3)	1
Mechanical ventilation (yes (%))	650 (62.6)	295 (55.3)	355 (70.2)	<0.001
Cause of discharge (%)				<0.001
Voluntary discharge	1 (0.1)	0 (0.0)	1 (0.2)	
Home	816 (78.5)	476 (89.3)	340 (67.2)	
Death	120 (11.5)	15 (2.8)	105 (20.8)	
Health center transfer	31 (3.0)	3 (0.6)	28 (5.5)	
Hospital transfer	33 (3.2)	19 (3.6)	14 (2.8)	
Not registered	38 (3.7)	20 (3.8)	18 (3.6)	
CCI	3.58 (2.53)	1.58 (1.11)	5.68 (1.81)	<0.001
Myocardial infarction (yes (%))	79 (7.6)	3 (0.6)	76 (15.0)	<0.001
Congestive heart failure (yes (%))	54 (5.2)	1 (0.2)	53 (10.5)	<0.001
Peripheral vascular disease (yes (%))	32 (3.1)	0 (0.0)	32 (6.3)	<0.001
Cerebrovascular accident (yes (%))	22 (2.1)	1 (0.2)	21 (4.2)	<0.001
Dementia (yes (%))	42 (4.0)	1 (0.2)	41 (8.1)	<0.001
COPD (yes (%))	131 (12.6)	30 (5.6)	101 (20.0)	<0.001
Connective tissue disease (yes (%))	13 (1.3)	4 (0.8)	9 (1.8)	0.226
Peptic ulcer disease (yes (%))	2 (0.2)	0 (0.0)	2 (0.4)	0.456
Liver disease (yes (%))	35 (3.4)	2 (0.4)	33 (6.5)	<0.001
Diabetes mellitus (yes (%))	194 (18.7)	36 (6.8)	158 (31.2)	<0.001
Hemiplegia (yes (%))	2 (0.2)	1 (0.2)	1 (0.2)	1
Moderate to severe CKD (yes (%))	153 (14.7)	4 (0.8)	149 (29.4)	<0.001
Solid tumor (yes (%))	44 (4.2)	1 (0.2)	43 (8.5)	<0.001
Lymphoma (yes (%))	16 (1.5)	0 (0.0)	16 (3.2)	<0.001
Leukemia (yes (%))	8 (0.8)	0 (0.0)	8 (1.6)	0.01
AIDS (yes (%))	2 (0.2)	0 (0.0)	2 (0.4)	0.456

*p*-value: *t*-test for continuous variables and chi-square for categorical variables. ICU: intensive care unit; CCI: Charlson comorbidity index; COPD: chronic obstructive pulmonary disease; CKD: chronic kidney disease; AIDS: acquired immune deficiency syndrome.

**Table 2:** Baseline and outcome characteristics of COVID-19 patients from DATA SAVE LIVES categorized by cluster.

	Stratified by Cluster			
	A	B	C	*p*
n	496	403	147	
Age	66.1 (15.8)	66.1 (13.7)	83.1 (9.9)	<0.001
Sex (male (%))	252 (50.8)	287 (71.2)	92 (62.6)	<0.001
Hospitalization (days)	7.6 (5.6)	13.7 (11.9)	10.1 (7.9)	<0.001
ICU stay (yes (%))	0.10 (1.57)	1.36 (5.08)	0.17 (1.51)	<0.001
Mechanical ventilation (yes (%))	258 (52.0)	287 (71.2)	112 (76.2)	<0.001
Cause of discharge (%)				<0.001
Voluntary discharge	0 (0.0)	1 (0.2)	0 (0.0)	
Home	433 (87.3)	313 (77.7)	75 (51.0)	
Death	8 (1.6)	58 (14.4)	55 (37.4)	
Health center transfer	18 (3.6)	3 (0.7)	10 (6.8)	
Hospital transfer	14 (2.8)	16 (4.0)	3 (2.0)	
Not registered	23 (4.6)	12 (3.0)	4 (2.7)	
CCI	3.2 (2.4)	3.1 (2.2)	6.2 (2.2)	<0.001
Myocardial infarction (yes (%))	40 (8.1)	17 (4.3)	22 (15.1)	<0.001
Congestive heart failure (yes (%))	19 (3.8)	11 (2.8)	24 (16.4)	<0.001
Peripheral vascular disease (yes (%))	18 (3.6)	3 (0.8)	11 (7.5)	<0.001
Cerebrovascular accident (yes (%))	8 (1.6)	5 (1.3)	9 (6.2)	0.001
Dementia (yes (%))	23 (4.6)	8 (2.0)	11 (7.5)	0.01
COPD (yes (%))	67 (13.5)	35 (8.8)	29 (19.9)	0.002
Connective tissue disease (yes (%))	8 (1.6)	1 (0.3)	4 (2.7)	0.041
Peptic ulcer disease (yes (%))	1 (0.2)	0 (0.0)	1 (0.7)	0.271
Liver disease (yes (%))	17 (3.4)	16 (4.0)	2 (1.4)	0.314
Diabetes mellitus (yes (%))	89 (18.0)	59 (14.8)	46 (31.5)	<0.001
Hemiplegia (yes (%))	1 (0.2)	1 (0.3)	0 (0.0)	0.837
Moderate to severe CKD (yes (%))	44 (8.9)	46 (11.6)	63 (43.2)	<0.001
Solid tumor (yes (%))	12 (2.4)	15 (3.8)	17 (11.6)	<0.001
Lymphoma (yes (%))	8 (1.6)	5 (1.3)	3 (2.1)	0.784
Leukemia (yes (%))	4 (0.8)	2 (0.5)	2 (1.4)	0.586
AIDS (yes (%))	0 (0.0)	2 (0.5)	0 (0.0)	0.199

*p*-value: ANOVA for continuous variables and chi-square for categorical variables. ICU: intensive care unit; CCI: Charlson comorbidity index; COPD: chronic obstructive pulmonary disease; CKD: chronic kidney disease; AIDS: acquired immune deficiency syndrome.

**Table 3:** Logistic regression model for the risk of death.

	OR (95% CI)		*p*	AUC
Model 1				0.801
Age-independent CCI	1.09	(0.97–1.21)	0.126	
Sex (male)	2.66	(1.69–4.25)	0.000	
Age	1.09	(1.07–1.11)	0.000	
Model 2				0.810
Age-independent CCI	1.10	(0.98–1.23)	0.087	
Oxygen saturation	0.94	(0.9–0.98)	0.007	
Temperature	1.12	(0.82–1.54)	0.469	
Sex (male)	2.55	(1.63–4.09)	0.000	
Age	1.09	(1.07–1.11)	0.000	
Model 3				0.871
Cluster (Cluster B)	12.83	(6.11–30.54)	0.000	
Cluster (Cluster C)	14.29	(6.66–34.43)	0.000	
Age-independent CCI	1.05	(0.93–1.18)	0.431	
Oxygen saturation	0.96	(0.92–1)	0.071	
Temperature	0.81	(0.58–1.13)	0.231	
Sex (male)	2.12	(1.31–3.52)	0.003	
Age	1.08	(1.06–1.11)	0.000	

OR: Odds Ratio; CI: Confidence interval; AUC: area under the curve; CCI: Charlson comorbidity index.

**Table 4:** Logistic regression model for the risk of mechanical ventilation.

	OR (95% CI)		*p*	AUC
Model 1				0.775
Age-independent CCI	1.18	(1.08–1.29)	0.000	
Sex (male)	1.17	(0.9–1.53)	0.246	
Age	1.02	(1.01–1.03)	0.000	
Model 2				0.749
Age-independent CCI	1.20	(1.1–1.32)	0.000	
Oxygen saturation	1.01	(0.98–1.05)	0.467	
Temperature	1.49	(1.22–1.83)	0.000	
Sex (male)	1.16	(0.88–1.51)	0.291	
Age	1.02	(1.01–1.03)	0.000	
Model 3				0.807
Cluster (Cluster B)	2.22	(1.64–3.01)	0.000	
Cluster (Cluster C)	1.71	(1.08–2.76)	0.024	
Age-independent CCI	1.21	(1.1–1.33)	0.000	
Oxygen saturation	1.02	(0.99–1.06)	0.205	
Temperature	1.28	(1.04–1.59)	0.021	
Sex (male)	1.00	(0.75–1.32)	0.980	
Age	1.02	(1.01–1.03)	0.000	

AUC: area under the curve; CCI: Charlson comorbidity index.

## Data Availability

HM Hospitales makes this clinical dataset available to researchers from academic, university and healthcare institutions who request it and whose project is approved. The content is expected to be expanded and updated periodically, and its update will not be completed until this pandemic is terminated. To obtain the data, it will be necessary to send the following request to the email coviddatasavelives@hmhospitales.com or data_science@hmhospitales.com in order to be evaluated by the Data Science Commission and, where appropriate, by the Research Ethics Committee of HM Hospitales or any other accredited research ethics committee.

## Supplementary Materials

The following supporting information can be downloaded at: https://www.mdpi.com/article/10.3390/jcm11123327/s1, Supplementary Table S1: Reference values for clinical variables included in the cluster analysis; Supplementary Table S2: Drug use by cluster and time (drugs with overall frequency n > 100); Supplementary Figure S1: A Principal component plot of the 2 main components from the cluster analysis; Supplementary Figure S2: Vital signs within the first 72 h of patients categorized by cluster; Supplementary Figure S3: White cells proportions within the first 72 h of patients categorized by cluster; Supplementary Figure S4: Red cells markers within the first 72 h of patients categorized by cluster; Supplementary Figure S5: Platelets and prothrombin markers within the first 72 h of patients categorized by cluster; Supplementary Figure S6: Metabolic markers and electrolytes within the first 72 h of patients categorized by cluster; Supplementary Figure S7: Inflammation and catabolic markers within the first 72 h of patients categorized by cluster.
