# Supplementary material for: Longwise Cluster Analysis for the Prediction of COVID-19 Severity within 72 h of Admission: COVID-DATA-SAVE-LIFES Cohort
Source: J Clin Med. 2022 Jun 10;11(12):3327. doi: 10.3390/jcm11123327 (PMC9224935; doi:10.3390/jcm11123327)
Supplement: Supplementary file 1 [file jcm-11-03327-s001.zip › jcm-1690968-supplementary.pdf]

Supplementary Table S1: Reference values for clinical variables included in the cluster analysis

	Reference range
Alanine aminotransferase (IU/L)	<33
Aspartate aminotransferase (IU/L)	<33
Basophil (%)	0.0 - 3.0
C-Reactive Protein (mg/L)	<10
Creatinine (mg/dL)	0.5 - 0.9
D-Dimer (ng/mL)	<500
Diastolic blood pressure (mmHg)	60 - 100
Eosinophils (%)	2.0 - 7.0
Gamma-glutamil transferasa (IU/L)	<60
Glucose (mg/dL)	70.0 - 105.0
Hematocrit (%)	35.0 - 47.0
Heart rate (beats/minunte)	60 - 100
Hemoglobin (g/dL)	12.3 - 15.3
International normalized ratio (INR)	0.8 - 1.2
Lactate Dehydrogenase (IU/L)	210.0 - 480.0
Leukocytes (1000/µL)	4.4 - 11.3
Lymphocyte (%)	20.0 - 50.0
Mean Corpuscular Hemoglobin (pg/cell)	28.0 - 33.0
Mean corpuscular hemoglobin concentration (g/dL)	33.0 - 36.0
Mean corpuscular volume (fL)	80.0 - 99.0
Mean platelet volume (%)	7.4 - 10.4
Monocyte (%)	2.0 - 8.0
Neutrophil (%)	40.0 - 75.0
Oxygen saturation (%)	94.0 - 100.0
Platelet count (1000/µL)	150.0 - 450.0
Potassium (mmol/L)	3.5 - 5.1
Prothrombin activity (%)	70.0 - 120.0
Prothrombin time (seconds)	9.9 - 14.2
Red Cell Distribution Width (%)	11.5 - 14.5
Sodium (mmol/L)	135.0 - 145.0
Systolic blood pressure (mmHg)	>130
Temperature (ºC)	36.0 - 36.9
Urea (mg/dL)	5.0 - 71.0

Supplementary Table S2: Drug use by cluster and time (drugs with overall frequency n>100)

	0hours				24hours				48hours				0hours			
	Cluster A	Cluster B	Cluster C	p	Cluster A	Cluster B	Cluster C	p	Cluster A	Cluster B	Cluster C	p	Cluster A	Cluster B	Cluster C	p
Agents against obstructive respiratory tract	32 (7.4)	35 (10.0)	4 (3.1)	**0.041**	34 (7.1)	55 (14.1)	13 (9.0)	**0.003**	12 (3.5)	16 (4.6)	6 (5.0)	0.674	7 (2.6)	7 (2.4)	2 (1.9)	0.915
Anti-inflammatory analgesics anti-rheumatic	20 (4.6)	23 (6.6)	3 (2.3)	0.146	24 (5.0)	23 (5.9)	11 (7.6)	0.488	14 (4.0)	16 (4.6)	4 (3.3)	0.818	6 (2.2)	23 (7.8)	8 (7.5)	**0.01**
Antipyretic analgesics	389 (89.8)	309 (88.5)	114 (89.1)	0.842	179 (37.4)	166 (42.6)	50 (34.7)	0.159	38 (11.0)	40 (11.5)	13 (10.7)	0.963	19 (7.1)	24 (8.1)	10 (9.3)	0.747
Angiotensin ii antagonists single drugs	39 (9.0)	11 (3.2)	19 (14.8)	**<0.001**	37 (7.7)	17 (4.4)	14 (9.7)	**0.042**	6 (1.7)	6 (1.7)	3 (2.5)	0.851	3 (1.1)	4 (1.4)	2 (1.9)	0.848
Serotonin 5 ht3 receptor antagonists	10 (2.3)	10 (2.9)	4 (3.1)	0.831	17 (3.6)	15 (3.8)	5 (3.5)	0.967	12 (3.5)	24 (6.9)	2 (1.7)	**0.023**	12 (4.5)	17 (5.7)	5 (4.7)	0.77
Antiasthmatic / bronchodilator	26 (6.0)	29 (8.3)	4 (3.1)	0.107	19 (4.0)	18 (4.6)	7 (4.9)	0.853	9 (2.6)	5 (1.4)	6 (5.0)	0.095	3 (1.1)	6 (2.0)	3 (2.8)	0.492
Antibiotics	272 (62.8)	227 (65.0)	67 (52.3)	**0.038**	245 (51.3)	234 (60.0)	94 (65.3)	**0.003**	48 (13.8)	65 (18.7)	35 (28.9)	**0.001**	31 (11.5)	41 (13.9)	15 (14.0)	0.668
Antidepressants	35 (8.1)	15 (4.3)	12 (9.4)	0.052	38 (7.9)	17 (4.4)	13 (9.0)	0.054	4 (1.2)	3 (0.9)	2 (1.7)	0.768	1 (0.4)	2 (0.7)	1 (0.9)	0.791
Antiemetic	39 (9.0)	36 (10.3)	10 (7.8)	0.67	39 (8.2)	28 (7.2)	14 (9.7)	0.621	22 (6.3)	23 (6.6)	6 (5.0)	0.808	15 (5.6)	23 (7.8)	3 (2.8)	0.165
Antipsychotics	10 (2.3)	7 (2.0)	7 (5.5)	0.094	21 (4.4)	17 (4.4)	23 (16.0)	**<0.001**	6 (1.7)	6 (1.7)	7 (5.8)	**0.024**	2 (0.7)	5 (1.7)	5 (4.7)	**0.034**
Antithrombotic antiagregant	176 (40.6)	143 (41.0)	48 (37.5)	0.777	203 (42.5)	168 (43.1)	72 (50.0)	0.262	47 (13.5)	54 (15.5)	25 (20.7)	0.175	22 (8.2)	24 (8.1)	14 (13.1)	0.259
Antiulcer gastric protector	203 (46.9)	177 (50.7)	56 (43.8)	0.338	171 (35.8)	134 (34.4)	65 (45.1)	0.064	32 (9.2)	40 (11.5)	17 (14.0)	0.306	21 (7.8)	17 (5.7)	5 (4.7)	0.442
Antivirals	21 (4.8)	20 (5.7)	5 (3.9)	0.697	13 (2.7)	29 (7.4)	3 (2.1)	**0.001**	2 (0.6)	8 (2.3)	2 (1.7)	0.166	2 (0.7)	1 (0.3)	1 (0.9)	0.726
HIV antivirals	64 (14.8)	117 (33.5)	15 (11.7)	**<0.001**	94 (19.7)	118 (30.3)	14 (9.7)	**<0.001**	30 (8.6)	49 (14.1)	6 (5.0)	**0.007**	19 (7.1)	29 (9.8)	3 (2.8)	0.059
Corticoids	26 (6.0)	35 (10.0)	13 (10.2)	0.082	24 (5.0)	81 (20.8)	31 (21.5)	**<0.001**	18 (5.2)	88 (25.3)	18 (14.9)	**<0.001**	15 (5.6)	75 (25.3)	23 (21.5)	**<0.001**
Antihypertensive diuretics	97 (22.4)	46 (13.2)	42 (32.8)	**<0.001**	91 (19.0)	66 (16.9)	52 (36.1)	**<0.001**	33 (9.5)	17 (4.9)	16 (13.2)	**0.007**	17 (6.3)	15 (5.1)	15 (14.0)	**0.007**
Hydroxychloroquine	205 (47.3)	179 (51.3)	48 (37.5)	**0.028**	187 (39.1)	164 (42.1)	59 (41.0)	0.677	30 (8.6)	27 (7.8)	12 (9.9)	0.753	10 (3.7)	15 (5.1)	7 (6.5)	0.483
Hypnotic sedant	68 (15.7)	27 (7.7)	14 (10.9)	**0.003**	63 (13.2)	53 (13.6)	25 (17.4)	0.433	27 (7.8)	38 (10.9)	3 (2.5)	**0.013**	15 (5.6)	29 (9.8)	8 (7.5)	0.171
Statin hypolipemian	43 (9.9)	19 (5.4)	13 (10.2)	0.053	24 (5.0)	19 (4.9)	17 (11.8)	**0.006**	3 (0.9)	5 (1.4)	1 (0.8)	0.733	3 (1.1)	1 (0.3)	1 (0.9)	0.544
Thyroid hormones	21 (4.8)	12 (3.4)	4 (3.1)	0.516	20 (4.2)	16 (4.1)	13 (9.0)	**0.041**	7 (2.0)	3 (0.9)	2 (1.7)	0.442	2 (0.7)	3 (1.0)	0 (0.0)	0.579
Aggregation inhibitors d3	34 (7.9)	12 (3.4)	10 (7.8)	**0.027**	27 (5.6)	15 (3.8)	14 (9.7)	**0.031**	4 (1.2)	4 (1.1)	2 (1.7)	0.898	1 (0.4)	2 (0.7)	0 (0.0)	0.649
Pirazolones	80 (18.5)	76 (21.8)	11 (8.6)	**0.004**	90 (18.8)	82 (21.0)	22 (15.3)	0.315	40 (11.5)	47 (13.5)	7 (5.8)	0.072	21 (7.8)	30 (10.1)	12 (11.2)	0.495
Tocilizumab	1 (0.2)	19 (5.4)	2 (1.6)	**<0.001**	2 (0.4)	26 (6.7)	7 (4.9)	**<0.001**	2 (0.6)	39 (11.2)	8 (6.6)	**<0.001**	4 (1.5)	38 (12.8)	3 (2.8)	**<0.001**

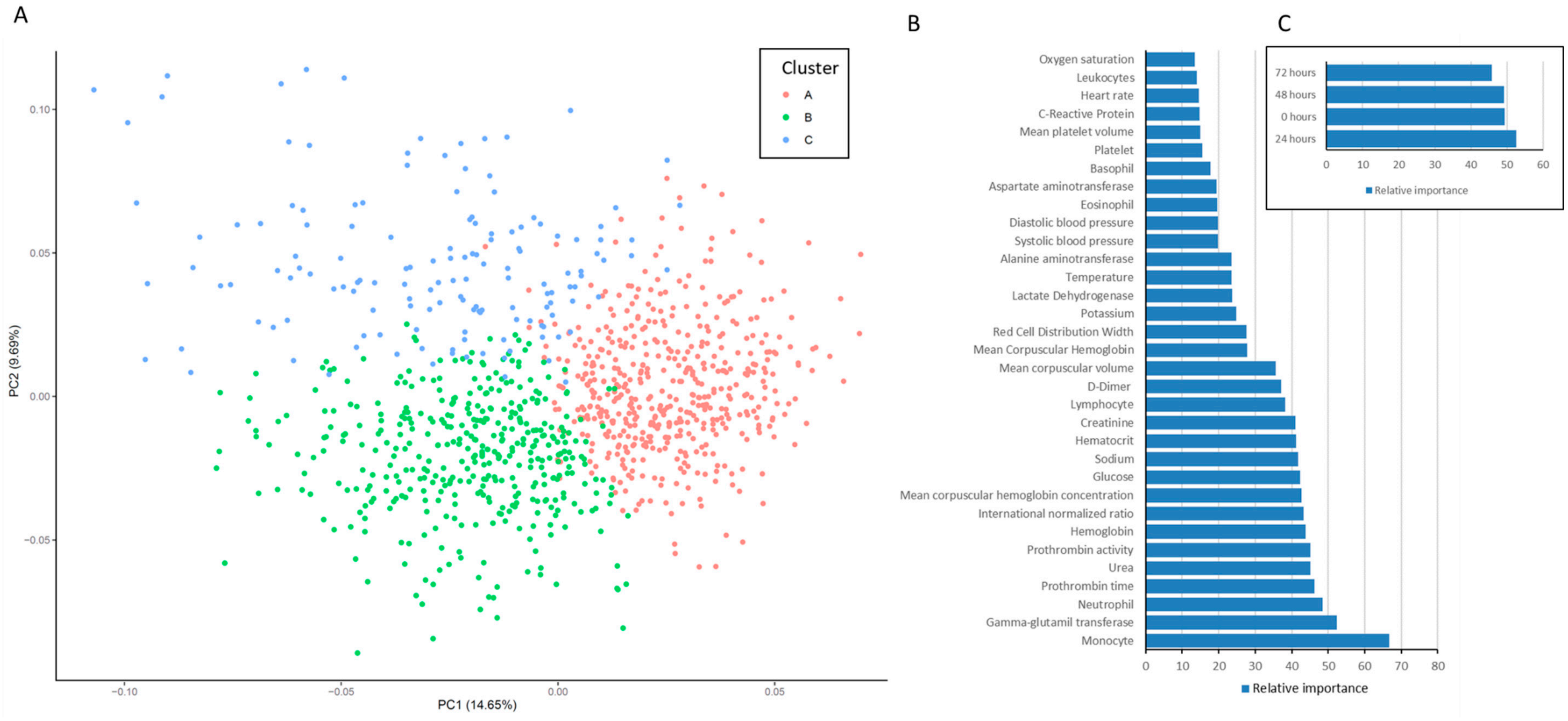

Supplementary Figure S1: A Principal component plot of the 2 main components from the cluster analysis. The first component decomposes the Cluster A (positive) and Clusters B & C (negative), while the second component splits the Cluster B (negative) and Cluster C (positive). **B:** Percentage of relative importance of input variables used for cluster classification. **C**: Percentage of relative importance of input blocks of 24 hours used for cluster classification. The B & C plot shows the average relative importance from the iterations of the final cluster solution.

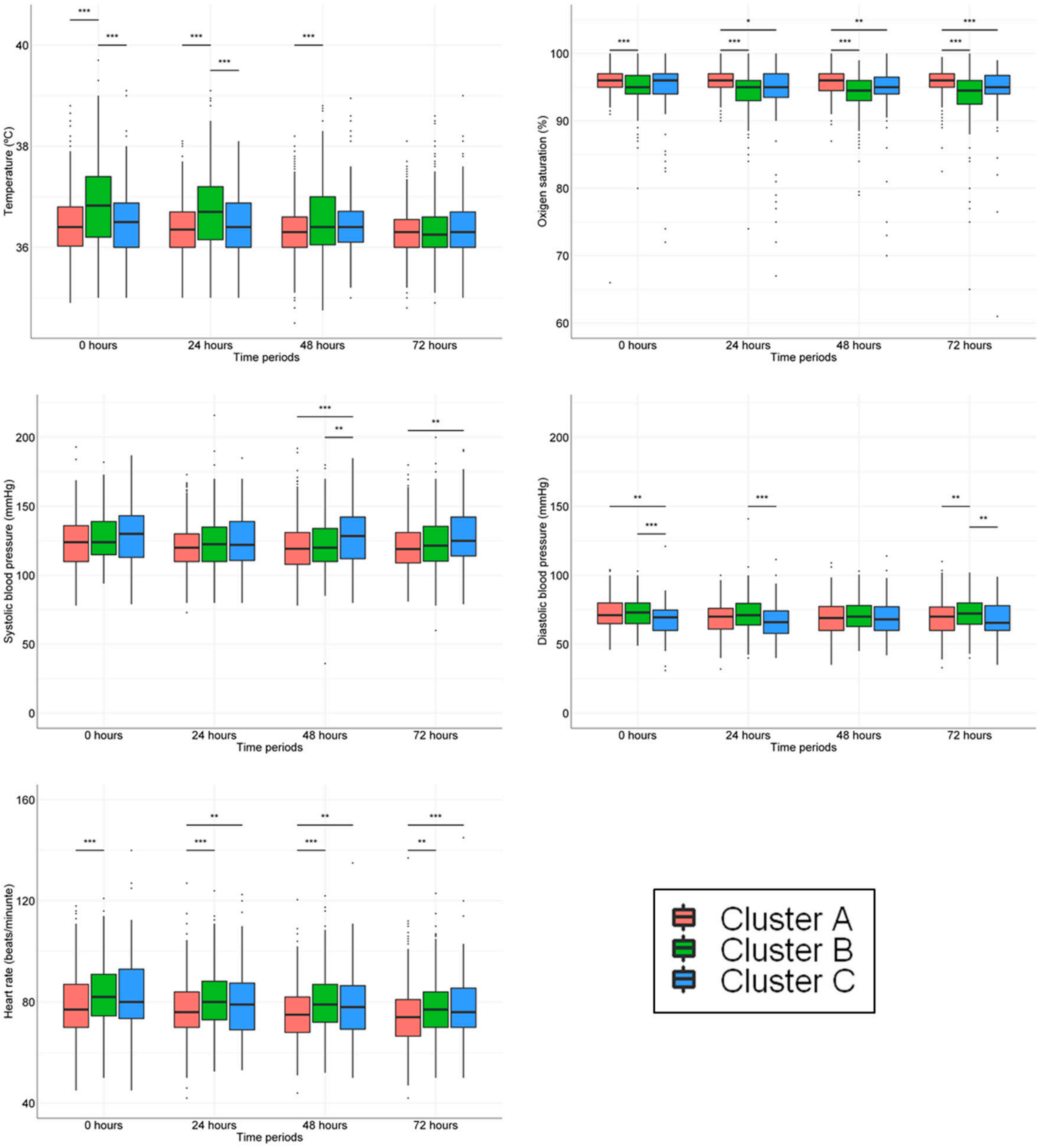

Supplementary Figure S2: Vital signs within the first 72h of patients categorized by cluster

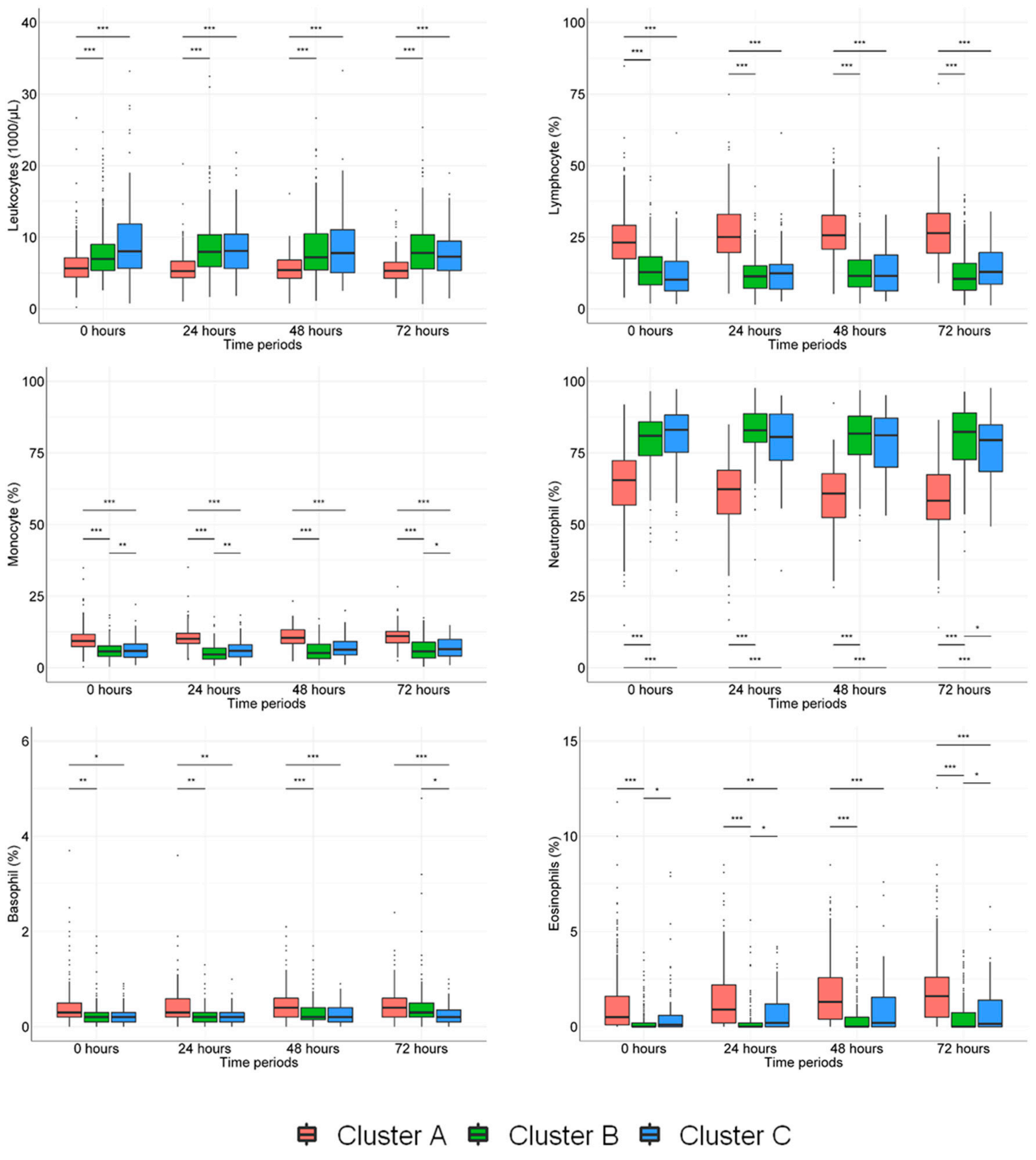

Supplementary Figure S3: White cells proportions within the first 72h of patients categorized by cluster.

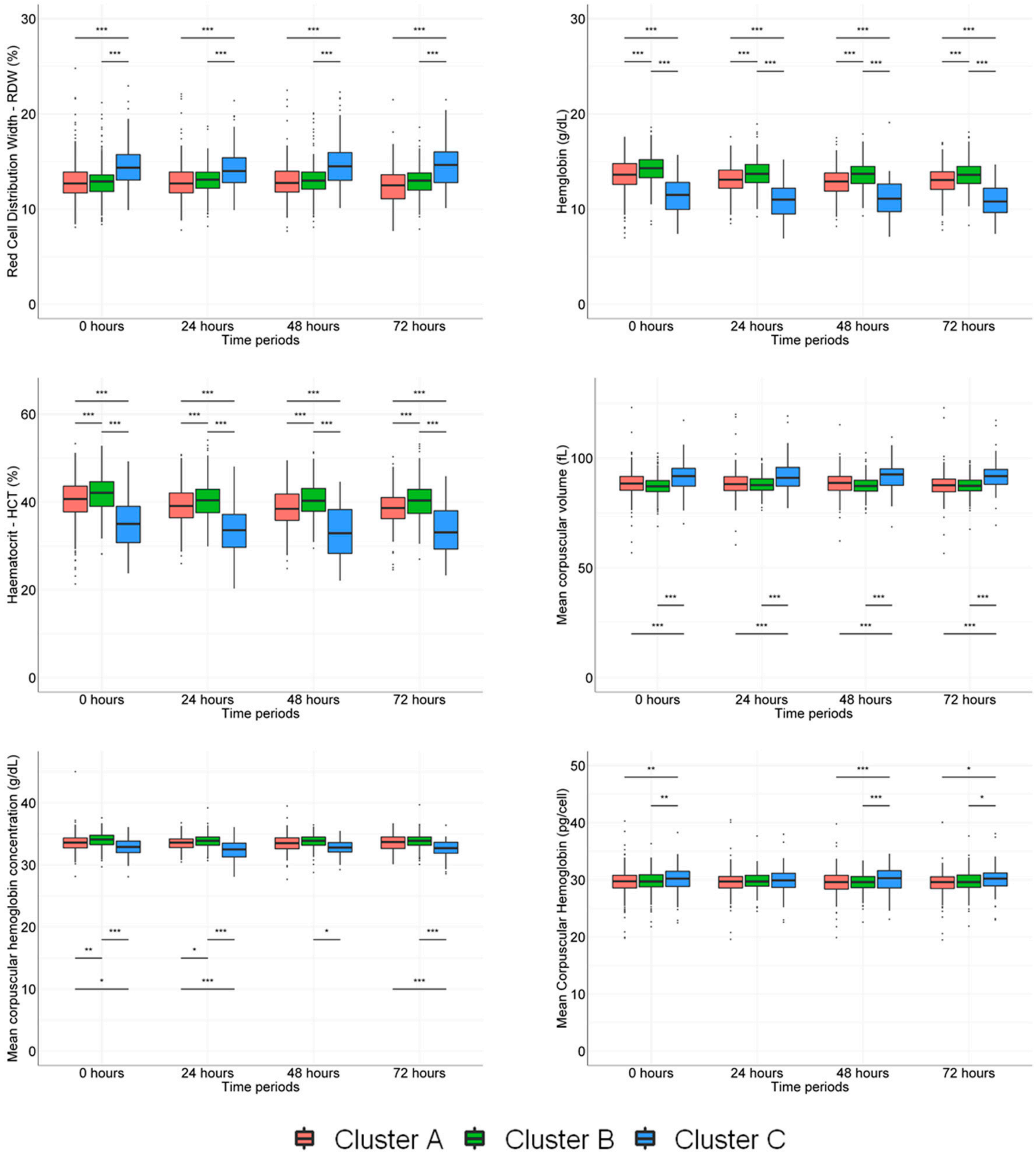

Supplementary Figure S4: Red cells markers within the first 72h of patients categorized by cluster.

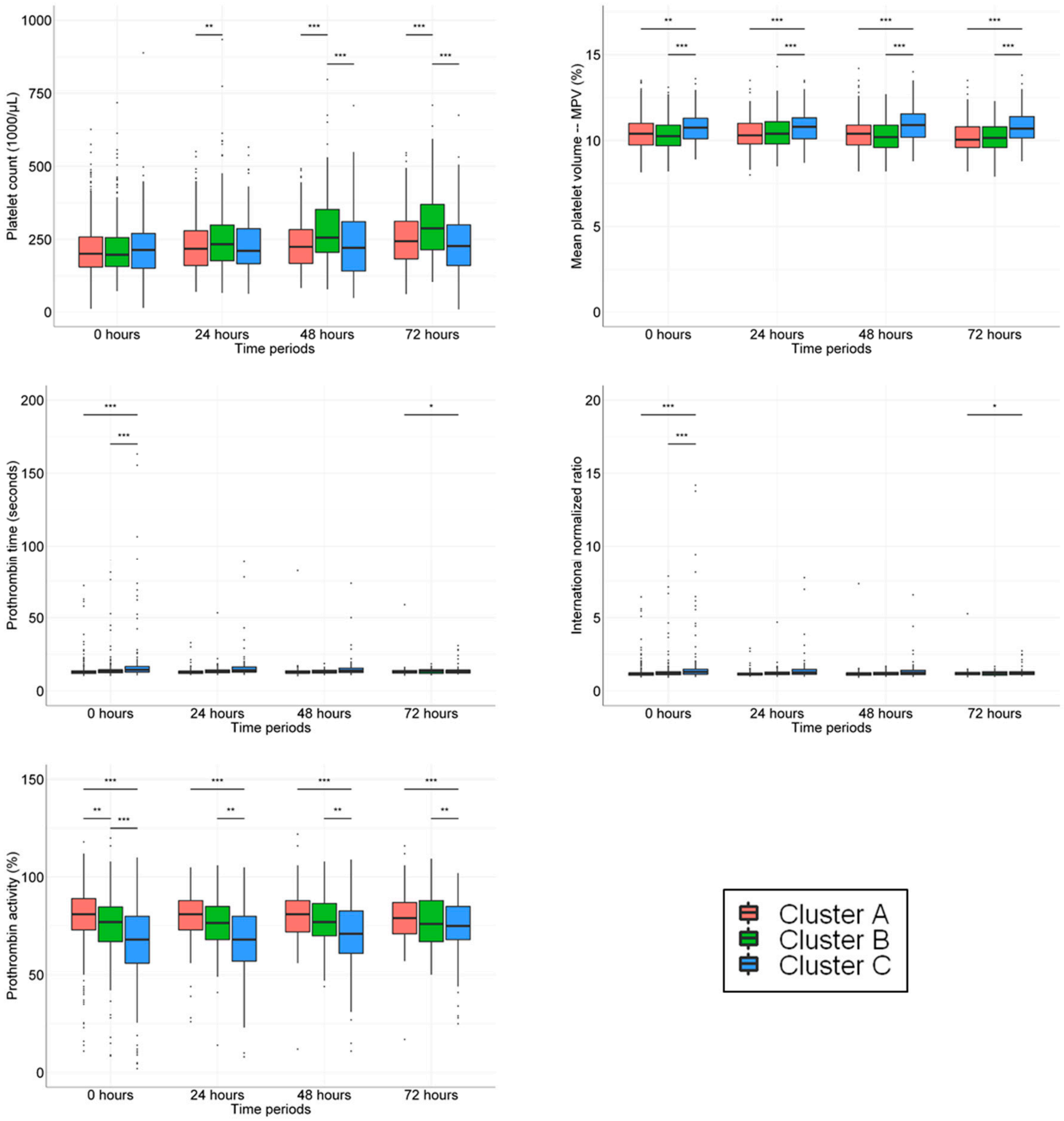

Supplementary Figure S5: Platelets and prothrombin markers within the first 72h of patients categorized by cluster.

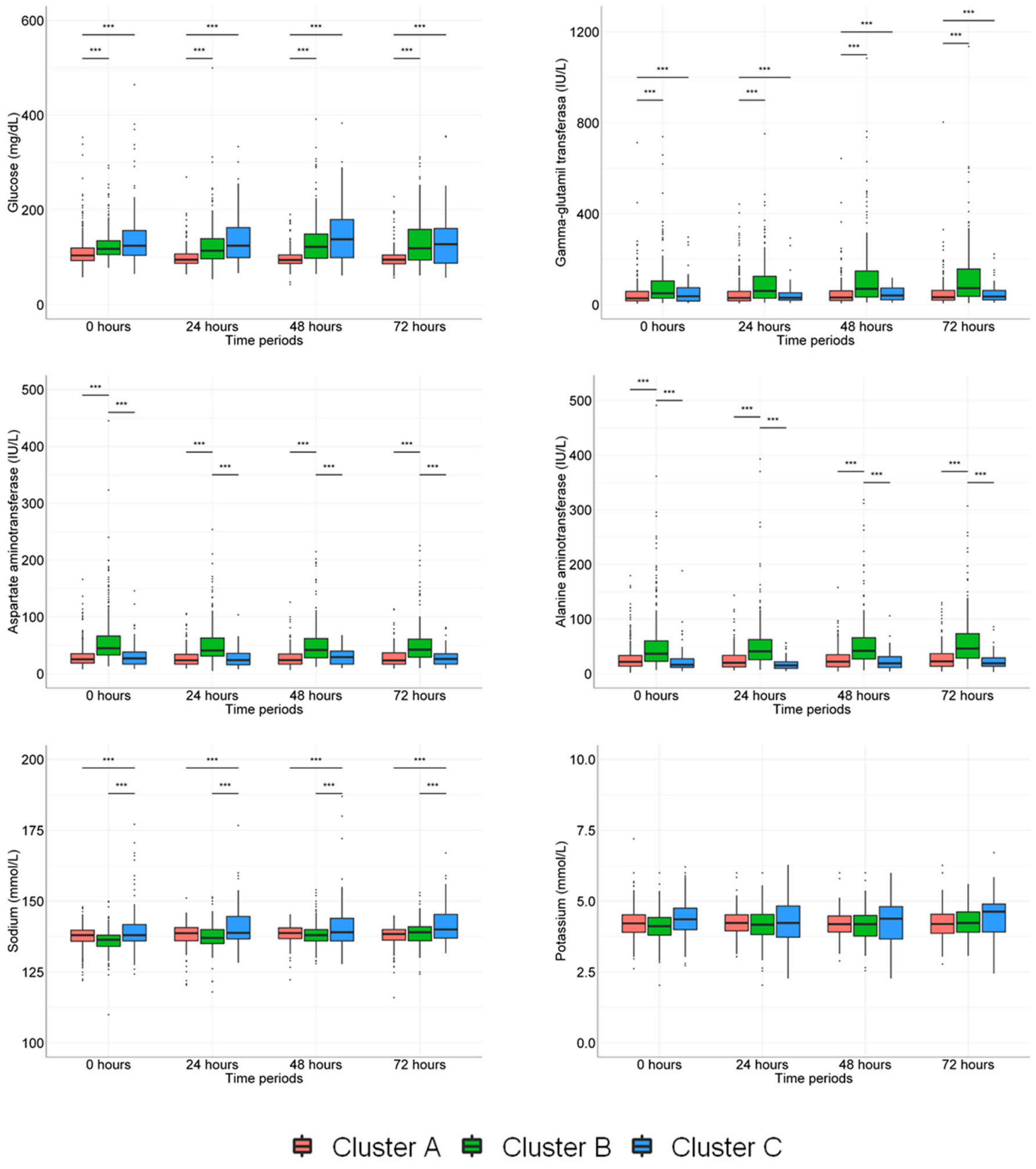

Supplementary Figure S6: Metabolic markers and electrolytes within the first 72h of patients categorized by cluster.

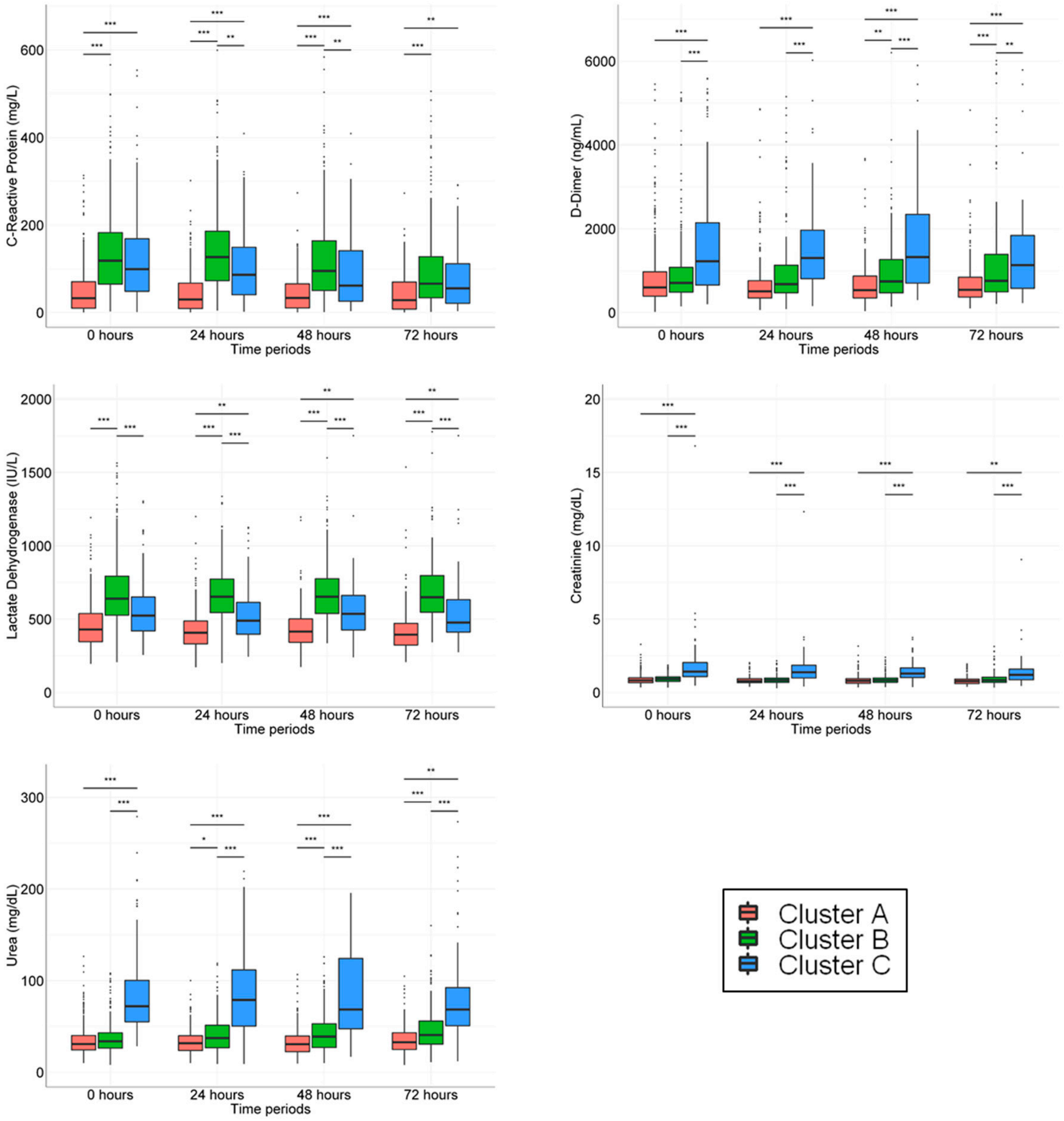

Supplementary Figure S7: Inflammation and catabolic markers within the first 72h of patients categorized by cluster.
